# H_2_S protects against fatal myelosuppression by promoting the generation of megakaryocytes/platelets

**DOI:** 10.1186/s13045-016-0244-7

**Published:** 2016-02-24

**Authors:** Huan-Di Liu, Ai-Jie Zhang, Jing-Jing Xu, Ying Chen, Yi-Chun Zhu

**Affiliations:** Shanghai Key Laboratory of Bioactive Small Molecules and Research Center on Aging and Medicine, Department of Physiology and Pathophysiology, Fudan University Shanghai Medical College, 138 Yi Xue Yuan Road, Shanghai, 200032 China; Collaborative Innovation Center of Molecular Diagnosis and Laboratory Medicine in Henan Province, School of Laboratory Medicine, Xinxiang Medical University, Xinxiang, China; Department of Pathology, The First Affiliated Hospital of Zhengzhou University, Zhengzhou, China

**Keywords:** Hydrogen sulfide, Myelosuppression, Hematopoiesis

## Abstract

**Background:**

Our previous pilot studies aimed to examine the role of hydrogen sulfide (H_2_S) in the generation of endothelial progenitor cells led to an unexpected result, i.e., H_2_S promoted the differentiation of certain hematopoietic stem/progenitor cells in the bone marrow. This gave rise to an idea that H_2_S might promote hematopoiesis.

**Methods:**

To test this idea, a mice model of myelosuppression and cultured fetal liver cells were used to examine the role of H_2_S in hematopoiesis.

**Results:**

H_2_S promoted the generation of megakaryocytes, increased platelet levels, ameliorate entorrhagia, and improved survival. These H_2_S effects were blocked in both in vivo and in vitro models with thrombopoietin (TPO) receptor knockout mice (c-mpl^−/−^ mice). In contrast, H_2_S promoted megakaryocytes/platelets generation in both in vivo and in vitro models with TPO knockout mice (TPO^−/−^ mice).

**Conclusions:**

H_2_S is a novel promoter for megakaryopoiesis by acting on the TPO receptors but not TPO to generate megakaryocytes/platelets.

**Electronic supplementary material:**

The online version of this article (doi:10.1186/s13045-016-0244-7) contains supplementary material, which is available to authorized users.

## Background

Serious myelosuppression is one of the fatal diseases manifested as a suppression on all types of hematopoietic cells and remains as one of the most difficult diseases to treat to date. Myelosuppression is either primary including aplastic anemia (AA) and myelodysplastic syndrome (MDS) or secondary to various disease status including chemotherapy and accidental/therapeutic radiation exposure [1–3]. Among the severe complications of myelosuppression, thrombocytopenia is immediately life threatening. Platelet transfusion is currently applied to treat severe thrombocytopenia; however, it has various side effects being common with the transfusion of blood components, and the endogenous generation of platelet is not ameliorated with such therapy [4, 5]. A more etiological treatment is the administration of recombinant thrombopoietin (TPO) which exerts significant effects in promoting platelet generation; however, it also has some side effects such as induction of autoantibodies due to the cross-reactions with endogenous TPO [4, 6, 7]. Since the effects of TPO receptor agonists are mild [6, 8, 9], exploration for new approaches to treat severe myelosuppression has potential translational values.

TPO has been established as a main cytokine to promote the generation of megakaryocytes and platelets by acting on its receptor, c-mpl [10–12]. The most established role of TPO is to promote the generation and differentiation of the hematopoietic stem cells in the bone marrow by acting on the TPO receptors, particularly the megakaryocytic stem/progenitor cells differentiated to megakaryocytes [13–15]. The TPO receptors are also found in the leukocytic and erythrocytic hematopoietic stem cells to be involved in the generation of leukocytes and erythrocytes in adulthood bone marrow [14, 16, 17].

Hydrogen sulfide (H_2_S) is endogenously generated in mammals via the H_2_S-generating enzymes cystathionine γ-lyase (CSE), cystathionine β-synthase (CBS), and 3-mercaptopyruvate sulfurtransferase (3-MST) [18–20]. H_2_S was considered as a metabolite without any physiological roles at the very start. The first piece of evidence reflecting a physiological role of H_2_S was found in organ bath experiments where an H_2_S donor, sodium hydrosulfide (NaHS), caused relaxation of the isolated vessels [21]. In the following years, H_2_S has been found to regulate the function of the cardiovascular system [22–24], the nervous system [25–27], the endocrine system [28, 29], and the immune system [30–32], among which the cardiovascular role of H_2_S is the most well established, including a regulation of vascular tone [33, 34] and remodeling [35–37], a protection against ischemia/reperfusion injury of the cardiomyocytes and myocardium [38, 39], regulation of ion channels [22, 40], and promotion of angiogenesis [41–43]. To date, any potential role of H_2_S in hematopoiesis is unknown.

Here, we report a novel protective effect of the gas molecule, H_2_S, against fatal myelosuppression being dependent on c-mpl. This is an unexpected finding in our studies investigating the role of endothelial progenitor cells in the proangiogenic effects of H_2_S which we found previously [41, 44, 45]. Though our data showed that the endothelial progenitor cells were not involved in these H_2_S effects, these pilot experiments revealed a novel promoting effect of H_2_S on hematopoiesis. This prompts us to propose a hypothesis that H_2_S may ameliorate myelosuppression.

In the present study, an H_2_S donor (NaHS) was applied to treat fatal myelosuppression in a mice model exposed to radiation and this is the first effort to examine the role of the gas transmitter H_2_S on hematopoiesis.

## Results

### H_2_S promotes megakaryocytes/platelets generation, alleviates bleeding, and improves survival in WT mice with radiation-induced myelosuppression

**Fig. 1:**
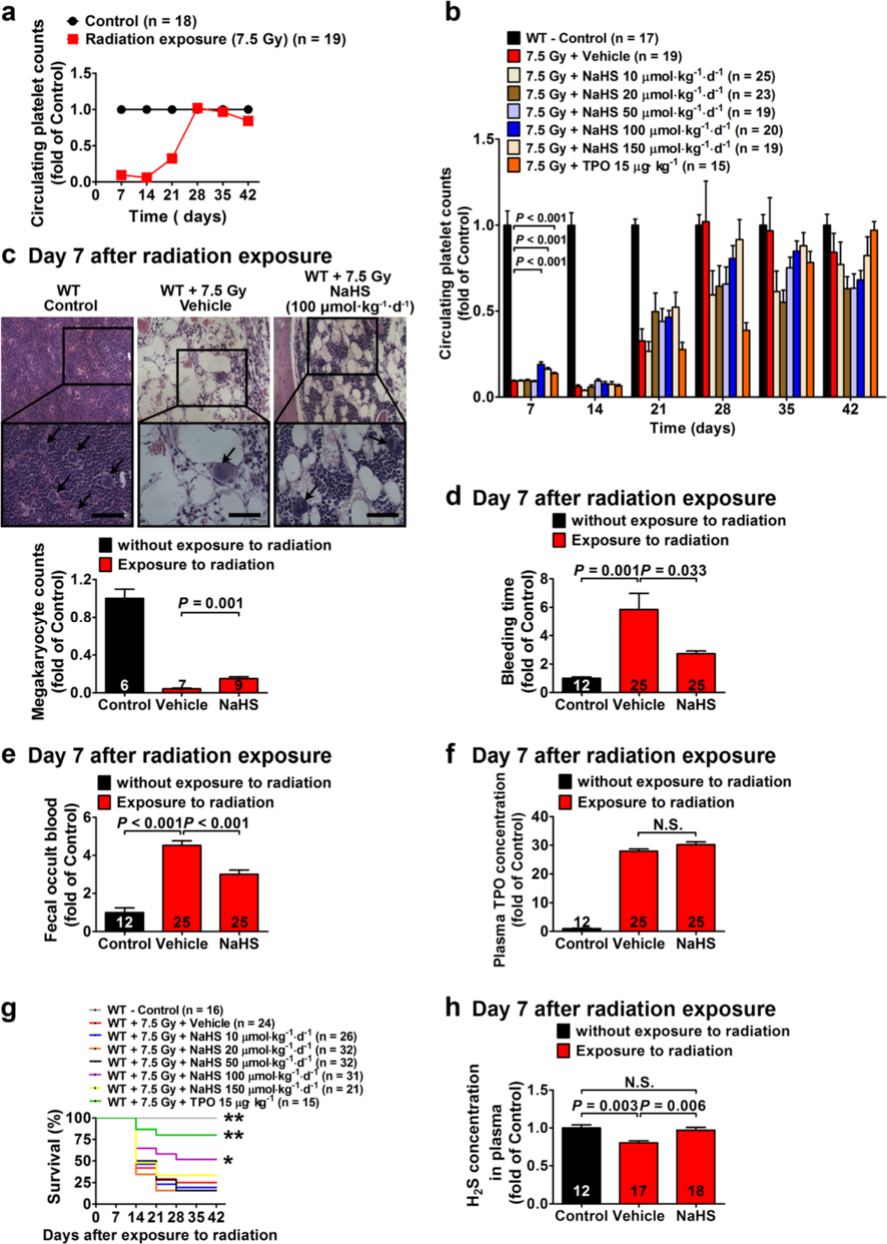
H_2_S treatment promotes platelet generation, alleviates bleeding, and improves survival in WT mice with radiation-induced myelosuppression. **a** Time course of circulating platelet counts after exposure to radiation of ^137^Cs at a single dose of 7.5 Gy. **b** NaHS treatment caused a significant increase in circulating platelet levels at 100 and 150 μmol kg^−1^ day^−1^ on day 7 after radiation exposure. TPO treatment also provided a similar amelioration at 15 μg kg^−1^. **c** Morphological examination showed a significant decrease in the number of megakaryocytes (*black arrow*) in the bone marrow of WT mice exposed to radiation, whereas NaHS (100 μmol kg^−1^ day^−1^) significantly increased the number of megakaryocytes as compared with the vehicle-treated group. *Scale bar* = 250 μm. **d** Bleeding time was prolonged after exposure to radiation, and bleeding was ameliorated by NaHS treatment (100 μmol kg^−1^ day^−1^) as compared with that of vehicle-treated WT mice. **e** Likewise, fecal occult blood was significantly increased after exposure to radiation and NaHS treatment (100 μmol kg^−1^ day^−1^) caused a significant decrease in fecal occult blood in mice exposed to radiation. **f** Plasma TPO concentrations were increased in WT mice on day 7 after radiation exposure where NaHS treatment (100 μmol kg^−1^ day^−1^) did not change TPO concentrations. **g** Radiation exposure resulted in a significant decrease in survival as compared with that of the mice without radiation exposure. Both NaHS (100 μmol kg^−1^ day^−1^) and TPO (15 μg kg^−1^) treatment significantly improved survival in these mice exposed to radiation. *P < 0.05, **P < 0.01 versus vehicle-treated mice exposed to radiation, log-rank test. **h** Plasma H_2_S levels were decreased in mice exposed to radiation, and this decrease was recovered by administration of NaHS (100 μmol kg^−1^ day^−1^). Data in the graphs are means ± SEM. *P* values less than 0.05 represent statistical significance

Exposure to radiation caused significant arrest of the bone marrow evidenced with a decrease in platelet levels in days 7, 14 and 21 in a mice model. The eliminating effect of radiation on circulating platelets peaked at days 7 and 14. Circulating platelet levels rebounded to control levels at day 28 and remained stable afterwards (Fig. 1a). Treatment with NaHS caused a significant increase in platelet levels at day 7 at dosages of 100 and 150 μmol kg^−1^ day^−1^ as compared with the vehicle-treated mice with radiation-induced myelosuppression (Fig. 1b). TPO treatment (15 μg kg^−1^) also increased platelet levels at day 7 (Fig. 1b). Morphological examination showed that NaHS treatment (100 μmol kg^−1^ day^−1^) significantly increased the number of megakaryocytes in the bone marrow of the mice exposed to radiation at day 7 (Fig. 1c). Bleeding time was approximately sixfold longer in the mice exposed to radiation at day 7, and NaHS treatment (100 μmol kg^−1^ day^−1^) significantly decreased bleeding time in the mice exposed to radiation (Fig. 1d). Likewise, fecal occult blood was significantly increased in the mice exposed to radiation as compared with those in the controls without exposure to radiation. NaHS treatment (100 μmol kg^−1^ day^−1^) significantly decreased fecal occult blood in the mice exposed to radiation (Fig. 1e). Plasma TPO levels were dramatically increased after radiation exposure; however, they were not changed by NaHS treatment (100 μmol kg^−1^ day^−1^) (Fig. 1f). Radiation exposure caused a mortality rate of 58 and 71 % at day 14 and day 21, respectively. Mortality did not further increase after day 28 in all groups. Survival was significantly increased in the mice treated with NaHS at a dosage of 100 μmol kg^−1^ day^−1^ at days 14, 21, 28, 35, and 42 after radiation exposure (*P* < 0.05 vs the vehicle-treated group) (Fig. 1g). TPO treatment (15 μg kg^−1^) also caused a significant increase in survival (Fig. 1g). On the other hand, endogenous H_2_S generation might be decreased after radiation exposure, since we found decreased plasma H_2_S levels after radiation exposure at day 7 (Fig. 1h), whereas NaHS treatment (100 μmol kg^−1^ day^−1^) recovered plasma H_2_S to a level comparable to that in the mice without radiation exposure (Fig. 1h).

### H_2_S promotes differentiation of the hematopoietic stem cells into megakaryocytes

**Fig. 2:**
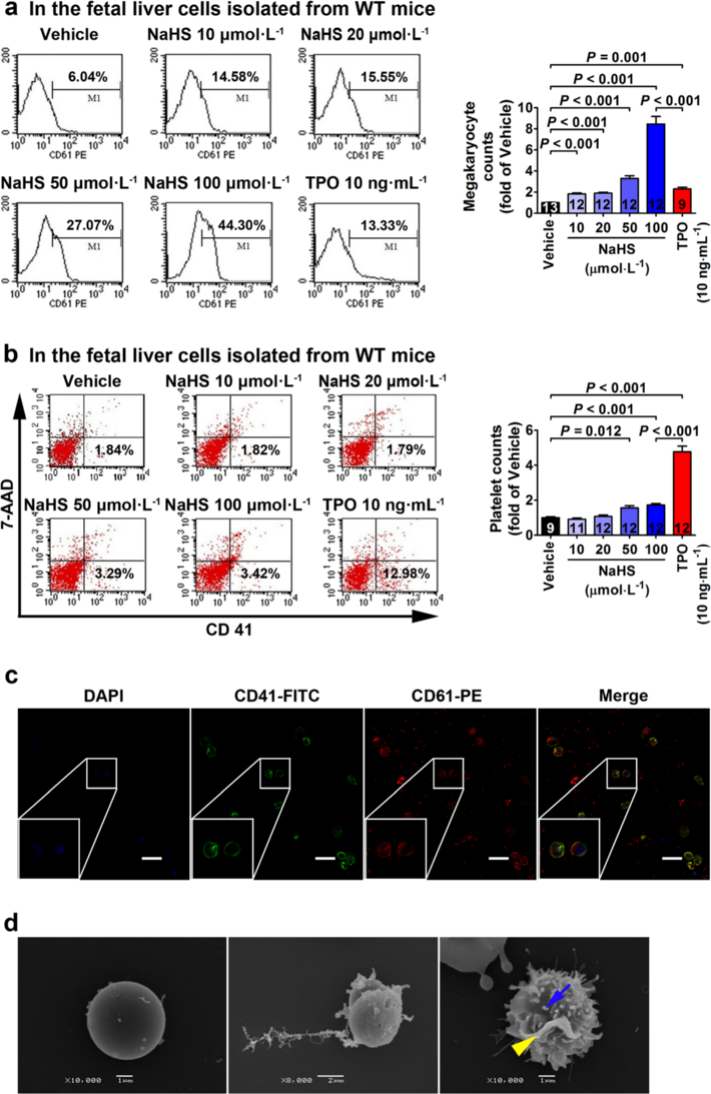
H_2_S treatment promotes generation of megakaryocytes/platelets in cultured fetal liver cells. **a** NaHS treatment caused a concentration-dependent increase in megakaryocytes which were identified as CD61-positive cells using flow cytometry in fetal liver cells isolated from WT mice. **b** Platelets were identified as CD41-positive and 7-AAD-negative signals using flow cytometry. Platelet counts were significantly increased with NaHS treatment at 50 and 100 μmol L^−1^ in the culture medium of fetal liver cells. TPO treatment (10 ng mL^−1^) also increased platelet counts. **c** Morphology of megakaryocytes in cultured fetal liver cells were shown with a confocal fluorescence microscopy with staining of DAPI, CD41-FITC, and CD61-PE. *Scale bar* = 50 μm. **d** Scanning electron microscopy revealed pseudopod formation (*blue arrow*) and membrane blebbing (*yellow arrow*) in the megakaryocytes identified in cultured fetal liver cells treated with NaHS (100 μmol L^−1^). Data in the graphs are means ± SEM. *P* values less than 0.05 represent statistical significance

To investigate if the abovementioned in vivo effect of H_2_S resulted from a stimulation on the hematopoietic stem cells, the potential effect of H_2_S on the differentiation of the hematopoietic stem cells into megakaryocytes was examined with flow cytometry where CD61 was used as the surface molecular marker for the megakaryocyte (Fig. 2a). These cells were further examined with confocal microscopy with double-staining using fluorescent-labeled antibodies against CD41 (green) and CD61 (red) where these two markers were co-localized on the cell membrane (Fig. 2c). This is in accordance with the literature that both CD41 and CD61 are expressed on the cell membrane of mature megakaryocytes. Since the hematopoietic stem cells are originally generated in fetal liver at the 11.5 day during the embryonic period (E11.5) and then expand at E14.5 before migration to the bone marrow [46, 47], we harvested the E14 fetal liver cells which were rich in hematopoietic stem cells to examine the in vitro effect of H_2_S in the present study. NaHS treatment concentration dependently promoted differentiation of cultured fetal liver cells into the megakaryocytes at 10, 20, 50, and 100 μmol L^−1^ as examined with flow cytometry (Fig. 2a). Platelet generation was also increased in the cultured fetal liver cells treated with NaHS at concentrations of 50 and 100 μmol L^−1^ examined with flow cytometry defined as CD41-positive and 7-AAD-negative signals, respectively (Fig. 2b). H_2_S-induced increase in megakaryocyte generation at a concentration of 100 μmol L^−1^ was even higher than that induced by TPO at 10 ng mL^−1^ (Fig. 2a), whereas TPO treatment (10 ng mL^−1^) showed a more significant effect in promoting platelet generation than NaHS treatment (100 μmol L^−1^) (Fig. 2b). Scanning electron microscopy showed the morphology of the megakaryocytes where pseudopod formation (blue arrow) and membrane blebbing (yellow arrow head) were observed in the cells treated with NaHS (Fig. 2d).

### c-mpl is required for H_2_S to promote megakaryocytes/platelets generation and improve survival

**Fig. 3:**
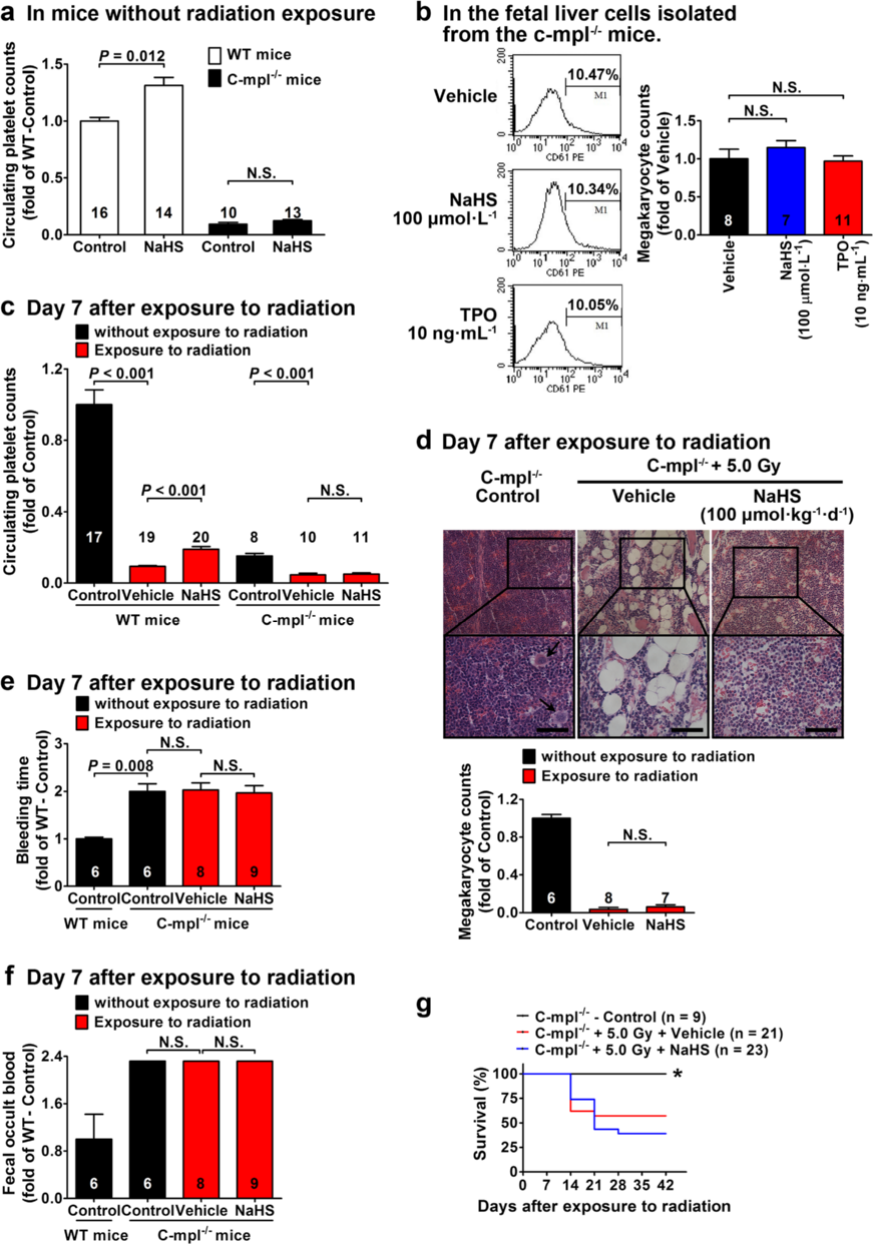
H_2_S-induced promotion of megakaryocytes/platelets generation and improvement of survival are blunted in c-mpl^−/−^ mice. **a** Without radiation exposure, NaHS treatment (20 μmol kg^−1^ day^−1^) for 14 days caused an increase in circulating platelet levels in WT mice but not in c-mpl^−/−^ mice. **b** Flow cytometry showed that NaHS treatment (100 μmol L^−1^) failed to increase the number of megakaryocytes identified as CD61-positive cells in cultured fetal liver cells isolated from c-mpl^−/−^ mice. **c** Circulating platelets were significantly decreased in both WT and c-mpl^−/−^ mice exposed to radiation at day 7. NaHS treatment (100 μmol kg^−1^ day^−1^) increased platelet levels in WT mice but not in c-mpl^−/−^ mice. **d** Morphological examination of the bone marrow showed that NaHS treatment (100 μmol kg^−1^ day^−1^) failed to increase the number of megakaryocytes (*black marrows*) in c-mpl^−/−^ mice. *Scale bar* = 250 μm. **e** Bleeding was not ameliorated with NaHS treatment (100 μmol kg^−1^ day^−1^) in c-mpl^−/−^ mice exposed to radiation at day 7. **f** Likewise, fecal occult blood was not ameliorated with NaHS treatment (100 μmol kg^−1^ day^−1^) in c-mpl^−/−^ mice exposed to radiation at day 7. **g** NaHS treatment (100 μmol kg^−1^ day^−1^) failed to improve survival in c-mpl^−/−^ mice exposed to radiation as compared to vehicle-treated mice. *P < 0.05 versus vehicle-treated mice exposed to radiation, log-rank test. Data in the graphs are means ± SEM. *P* values less than 0.05 represent statistical significance

To further clarify how H_2_S can promote megakaryocytes generation and provide protection against fatal myelosuppression, some knockout mice models were applied. TPO has a pivotal role in the generation of megakaryocytes and platelets by acting on its receptor, c-mpl [10–12]. The data showed that platelet count was decreased by about 80 % in c-mpl^−/−^ mice as compared with that in wild-type (WT) mice (Fig. 3a). NaHS treatment (20 μmol kg^−1^ day^−1^) for 14 days caused an increase in circulating platelet levels in WT mice but not in c-mpl^−/−^ mice (Fig. 3a). Likewise, NaHS treatment promoted the generation of megakaryocytes in the fetal liver cells isolated from WT mice at concentrations ranging from 10 to 100 μmol L^−1^ (Fig. 2a) but not in the fetal liver cells isolated from the c-mpl^−/−^ mice (Fig. 3b). Circulating platelet levels were significantly decreased in c-mpl^−/−^ mice as compared with those of the WT mice. Radiation exposure caused significant decrease in circulating platelet levels in both WT and c-mpl^−/−^ mice, and this decrease was partially recovered with NaHS treatment (100 μmol kg^−1^ day^−1^) in WT mice but not in c-mpl^−/−^ mice (Fig. 3c). Likewise, NaHS treatment increased megakaryocyte levels in the bone marrow in WT mice exposed to radiation (Fig. 1c) but not in those of the c-mpl^−/−^ mice (Fig. 3d). Bleeding time (Fig. 3e) and fecal occult blood (Fig. 3f) were not ameliorated by NaHS treatment (100 μmol kg^−1^ day^−1^) in the c-mpl^−/−^ mice exposed to radiation at day 7, though these parameters were improved in the WT mice by NaHS treatment (100 μmol kg^−1^ day^−1^) (Fig. 1d, e). Finally, NaHS treatment (100 μmol kg^−1^ day^−1^) improved survival after radiation exposure in WT mice (Fig. 1g) but not in c-mpl^−/−^ mice (Fig. 3g).

### The role of TPO in H_2_S-induced promotion on megakaryocytes/platelets generation

**Fig. 4:**
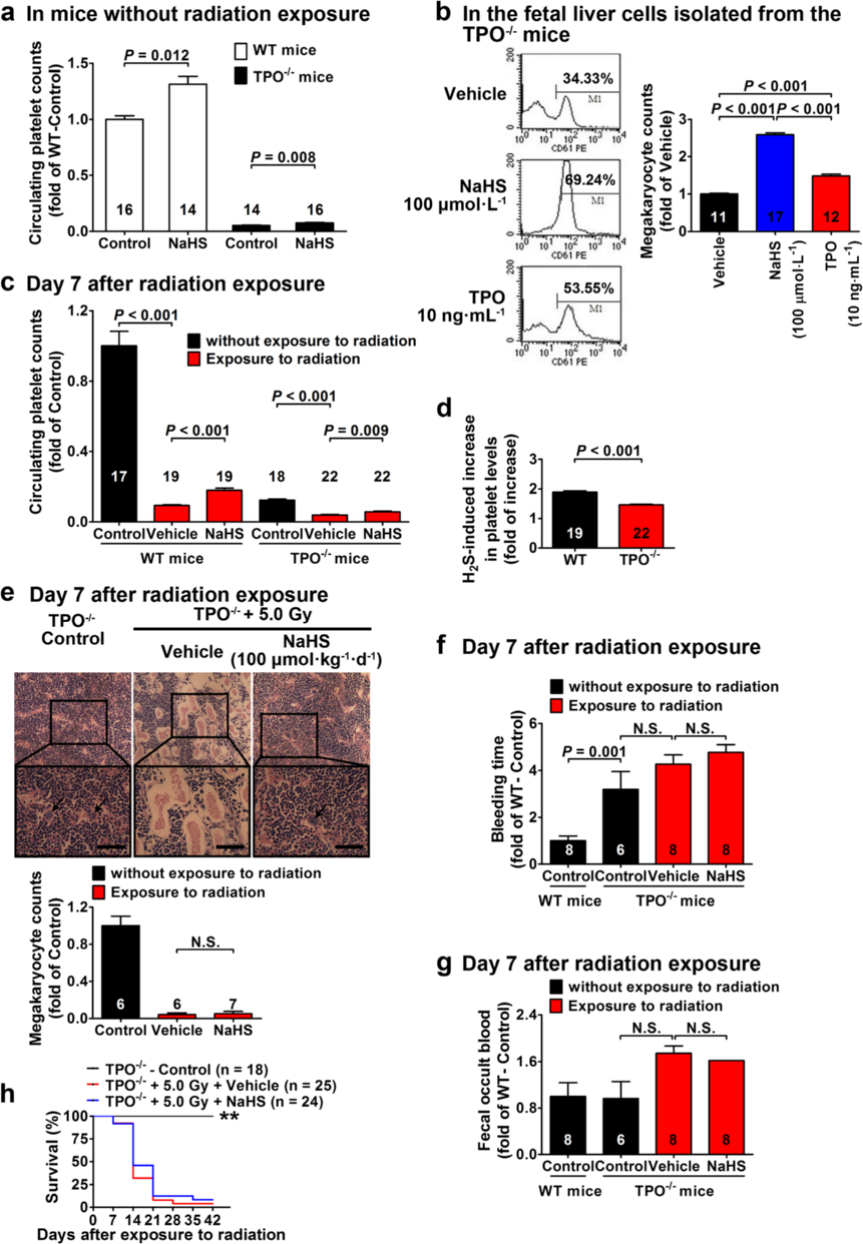
H_2_S promotes megakaryocytes/platelets generation but does not improve survival in TPO^−/−^ mice. **a** Without radiation exposure, NaHS treatment (20 μmol kg^−1^ day^−1^) for 14 days caused an increase in circulating platelet levels in both WT and TPO^−/−^ mice. **b** NaHS treatment (100 μmol L^−1^) increased the number of megakaryocytes identified as CD61-positive cells using flow cytometry in cultured fetal liver cells isolated from TPO^−/−^ mice. **c**, **d** NaHS treatment (100 μmol kg^−1^ day^−1^) increased circulating platelet levels on day 7 after radiation exposure in both WT and TPO^−/−^ mice (**c**). However, the effects of NaHS treatment (100 μmol kg^−1^ day^−1^) in increasing circulating platelet levels were blunted in TPO^−/−^mice (**d**). **e** Morphological examination of the bone marrow showed that NaHS treatment (100 μmol kg^−1^ day^−1^) did not increase the number of megakaryocytes (*black arrows*) in the TPO^−/−^ mice exposed to radiation. *Scale bar* = 250 μm. **f** Bleeding was not ameliorated with NaHS treatment (100 μmol kg^−1^ day^−1^) in the TPO^−/−^ mice exposed to radiation at day 7. **g** Likewise, fecal occult blood was not ameliorated with NaHS treatment (100 μmol kg^−1^ day^−1^) in the TPO^−/−^ mice exposed to radiation at day 7. **h** Survival was not improved with NaHS treatment (100 μmol kg^−1^ day^−1^) in TPO^−/−^ mice exposed to radiation. **P < 0.01 versus vehicle-treated mice exposed to radiation, log-rank test. Data in the graphs are means ± SEM. *P* values less than 0.05 represent statistical significance

On the other hand, TPO is the main cytokine to promote the production of megakaryocytes and platelets [11, 48, 49]. Though plasma TPO levels were not increased by H_2_S treatment in WT mice exposed to radiation (Fig. 1f), the role of TPO in mediating the hematopoietic effects of H_2_S was still worthy to be investigated. Therefore, the potential hematopoietic effects of H_2_S were also examined in TPO^−/−^ mice with or without radiation exposure and in the fetal liver cells isolated from the TPO^−/−^ mice. TPO knockout caused about 90 % reduction in circulating platelet levels in mice without radiation exposure (Fig. 4a). NaHS treatment (20 μmol kg^−1^ day^−1^) for 14 days caused about a 43 % increase in circulating platelet levels in TPO^−/−^ mice (Fig. 4a). Megakaryocyte generation was promoted with NaHS treatment (100 μmol L^−1^) in fetal liver cells isolated from the TPO^−/−^ mice, and this effect was more pronounced than that with exogenous TPO treatment (10 ng mL^−1^) (Fig. 4b). Radiation exposure caused a significant decrease in circulating platelet levels in both WT mice and TPO^−/−^ mice (Fig. 4c). NaHS treatment (100 μmol kg^−1^ day^−1^) increased circulating platelet levels in both WT mice and TPO^−/−^ mice exposed to radiation (Fig. 4c), whereas this H_2_S effect in TPO^−/−^ mice was less pronounced than that in WT mice (Fig. 4d). However, NaHS treatment (100 μmol kg^−1^ day^−1^) did not cause a significant increase in megakaryocyte levels in the bone marrow of the TPO^−/−^ mice exposed to radiation (Fig. 4e). Likewise, NaHS treatment (100 μmol kg^−1^ day^−1^) failed to ameliorate bleeding (Fig. 4f) and fecal occult blood (Fig. 4g) despite an increase in the circulating platelet levels in TPO^−/−^ mice with radiation exposure and treated with NaHS treatment (100 μmol kg^−1^ day^−1^). As a final consequence, NaHS treatment (100 μmol kg^−1^ day^−1^) did not improve survival in TPO^−/−^ mice exposed to radiation (Fig. 4h) although survival was improved in WT mice (Fig. 1g).

## Discussion

This is the first time that the gas transmitter H_2_S has been found to protect against fatal myelosuppression with improvement in bone marrow morphology, circulating platelet levels, and final survival. The increased survival in H_2_S-treated mice may be ascribed to the decreased internal bleeding. This idea was supported by less bleeding time and fecal occult blood found in these H_2_S-treated mice. Moreover, these effects were found at physiologically relevant dosages of H_2_S since the treatment just recovered the decrease in plasma H_2_S levels in radiation-exposed mice. Therefore, these findings have potential translational values and may be further explored for new approaches to treat fatal diseases resulting from severe myelosuppression.

In the embryonic period, hematopoiesis mainly occurs in the liver where the hematopoietic stem cells were differentiated into various types of blood cells [50–52]. Here, we show a novel protective role of H_2_S in fatal myelosuppression, i.e., H_2_S ameliorates internal bleeding and improves survival by promoting generation of megakaryocytes and platelets. Future works are required to further validate this hypothesis using multiple approaches including bone marrow transplantation experiments.

We found here that the hematopoietic effects of H_2_S were blunted in the c-mpl^−/−^ mice exposed to radiation and also in the fetal liver cells isolated from the c-mpl^−/−^ mice. These data suggest that the TPO receptors are essential for H_2_S to promote hematopoiesis. In the adult stage, the TPO receptors are expressed on different maturation stages of megakaryocyte and early hematopoietic stem/progenitor cells in the bone marrow [53–55]. The predominant role of the TPO receptors is to mediate the signals to induce proliferation and differentiation of megakaryocyte progenitor cells to generate megakaryocytes and platelets [10–12]. In addition, since the TPO receptors are also expressed on the hematopoietic stem/progenitor cells, it is also involved in the expansion of erythroid, granulocyte-macrophage, and megakaryocytic progenitor cells [14, 16, 17]. Although there are also some evidence showing expression of the TPO receptors in the tissues outside of the bone marrow, e.g., in placenta, fetal liver, fetal blood, cord blood, and peripheral blood, bone marrow is the main place of hematopoiesis in adulthood [51, 52, 56]. In this context, if the hematopoietic effect of H_2_S is dependent on the TPO receptors, these H_2_S effects are mediated via the TPO receptors located in the hematopoietic stem/progenitor cells in the bone marrow. Indeed, the absence of more direct evidence using bone marrow transplantation models is a limitation of our current study.

The mechanisms about how H_2_S would activate the TPO receptors are unknown. How a molecule as small as H_2_S targets its “receptors” is one of the most challenging questions in the field. Our previous studies aim to find that the “receptor” of H_2_S shows that VEGFR2 serves as a direct target molecule for H_2_S to induce angiogenesis. The study further shows that H_2_S targets a specific molecular switch, i.e., the Cys1024-Cys1045 motif, to regulate the conformation and function of VEGFR2 by sulfur-sulfur nucleophilic attack [44]. This suggests a new mechanism beyond the typical docking between a ligand and its receptor. Some theories of atomic biology involving molecular orbitals are involved in this H_2_S-induced regulation of VEGFR2. Interestingly, the TPO receptors and VEGFR2 belong to the receptor tyrosine kinase family. Some members of this kinase family, e.g., EGFR, have been recently found to contain an intracellular kinase domain similar to that of the VEGFR2 also with a molecular switch labile to H_2_S regulation [57]. This gives rise to an idea that the TPO receptors might also contain a similar intracellular kinase domain with a molecular switch sensitive to H_2_S regulation. Indeed, much future works are required to examine this hypothesis.

In TPO^−/−^ mice exposed to radiation, H_2_S also failed to ameliorate internal hemorrhage and improve survival despite a significant increase in circulating platelet levels. In addition, H_2_S promoted megakaryocytes/platelets generation in cultured fetal liver cells isolated from TPO^−/−^ mice but not in c-mpl^−/−^ mice. These data suggest an essential role of the TPO receptor but not TPO for H_2_S to promote hematopoiesis. The question is why H_2_S increased platelet levels in TPO^−/−^ mice without ameliorating bleeding and subsequent mortality? This discrepancy might be ascribed to a significantly decreased survival in TPO^−/−^ mice treated with vehicle as compared with that in c-mpl^−/−^ mice (*P* < 0.01, Additional file 1: Figure S1). Myelosuppression might be too severe to be recovered by H_2_S in these TPO^−/−^ mice, whereas the TPO receptors were present in the cells isolated from the TPO^−/−^ mice and might serve as a target molecule for H_2_S to promote hematopoiesis.

In addition, H_2_S also increased circulating leukocytes in WT mice exposed to radiation at day 14 though erythrocytes were not increased (Additional file 2: Figure S2). Our present data are not sufficient to clarify whether such an improvement in circulating leukocyte counting contributes to an increase in survival in mice exposed to radiation due to an improvement in the immune system.

## Conclusions

Taken together, treatment for fatal myelosuppression is proven a difficult challenge in medicine. Here, we report a novel protective role of H_2_S in a mice model of fatal myelosuppression. This finding has potential translational values which could be further explored to develop new approaches to treat fatal myelosuppression. Moreover, experiments using the models with knockout of the TPO receptors or TPO itself indicate that the hematopoietic effect of H_2_S is dependent on the TPO receptors but not on TPO. In addition, the endogenous generation of H_2_S was reduced in the myelosuppression model and a recovery of the H_2_S levels was associated with a protection against radiation exposure. These findings shed some light to uncovering a new mechanism to regulate hematopoiesis by a gas transmitter, H_2_S.

## Methods

### Animals

Wild-type C57BL/6 mice (WT mice) were purchased from the Department of Laboratory Animal Science of Fudan University and acclimatized for at least 2 weeks before experiments. c-mpl is the receptor of TPO; the *c-mpl* knockout mice (c-mpl^−/−^ mice) were provided by The Jackson Laboratory (Bar Harbor, ME, USA) with an 80 % decrease in the number of platelets in comparison to that of wild-type C57BL/6 mice [58, 59] (the strain name of these c-mpl^−/−^ mice is C57BL/6J-*Mpl*^*hlb219*^/J with the stock number 005124). Likewise, TPO-deficient mice (TPO^−/−^ mice) have a >80 % decrease in their platelets [59, 60]. The embryos of TPO^−/−^ mice were gifts kindly provided by Dr. Fred de Sauvage (Genentech Inc., USA) and developed into mice through the Embryo Recovery Service of The Jackson Laboratory (these TPO^−/−^mice were produced by targeted mutation on the background of C57BL/6J mice; for more details, see de Sauvage [60]). Both TPO^−/−^ and c-mpl^−/−^ mice were hybridized with WT mice and then their offspring were genotyped according to the protocol provided by The Jackson Laboratory. Homozygote animals were used in the experiments while heterozygote mice were maintained for breeding. Only female mice were used in the experiments. Mice were given free access to food and water while housed in a clean environment with appropriate temperature and humidity. At the time of experiments, all mice were 6–8 weeks old weighting 18–21 g. Considering that in the experiment mice will die or suffer severe sickness in the process, we set at least 10 mice per group at the beginning and repeated the experiment if necessary. To ensure the reliability of results, mice were randomly allocated to each group. All procedures were approved by the Institutional Animal Care and Use Committee, Fudan University Shanghai Medical College (20120302-098).

### Irradiation-induced myelosuppression

The Cs-137 instrument (Gammacell-40, MDS Nordion, CA) was used as the γ-ray irradiation source in this study. Six- to 8-week-old mice were either exposed to 7.5 Gy (WT mice) or 5.0 Gy (TPO^−/−^ and c-mpl^−/−^ mice) cesium (^137^Cs)-emitted radiation for total body irradiation (TBI) to build up the myelosuppression model. The TPO^−/−^ and c-mpl^−/−^ mice were more sensitive to radiation exposure, and the mortality was too high to perform subsequent experiments in these mice. We performed a pilot study to explore the intensity of radiation exposure in these mice to cause radiation-induced myelosuppression with a mortality within the range that enables the subsequent experiments to be performed. The results showed 5.0 Gy as the radiation intensity for the TPO^−/−^ and c-mpl^−/−^ mice.

### Platelet counts

Platelet counts were measured by automatic blood cell analyzer (XN-1000, Sysmex, Kobe, Japan). About 80–100 μL peripheral blood was obtained from the angular vein of individual mice then pumped into a 1.5-mL Eppendorf tube coated with ethylenediaminetetraacetic acid (EDTA) and mixed immediately. For platelet counting, whole blood was used without any dilution. During all experiments, every single mouse was sampled at 7-day intervals. Frequency and timing of sampling of each individual mouse was identical in all types of mice in this study. In this part, the operator of the automatic blood analyzer was blinded to the sample groups. Results were excluded in cases where there was clotting in the sample.

### Bleeding time test

The tails of mice were placed horizontally then amputated about 3 mm from the tip and immersed into saline solution pre-warmed and maintained at 37 °C. The time from severing to cessation of bleeding was recorded as bleeding time, and the longest observation time was 20 min to avoid over bleeding which frequently occurred in the TPO^−/−^ and c-mpl^−/−^ mice. Researchers were blinded to the sample groups.

### Fecal occult blood test

A fecal occult blood test kit (Baso, Zhuhai, China) was used to examine occult gastrointestinal bleeding according to protocols provided by the manufacturer. Briefly, 20–30 mg feces were smeared on the window of the test card then dripped with a drop of developer A (pyramidon). After sufficient penetration, a drop of developer B (alcohol and hydrogen peroxide) was dripped and then the results were read by comparing with a color card within 2 min. Researchers were blinded to the sample groups.

### Bone marrow megakaryocytes counts

The femur and tibia were collected from some mice sacrificed at day 7 during the experimental period. After fixing in 10 % formaldehyde and dehydrated, the bone samples were embedded into paraffin, sliced, and stained with hematoxylin and eosin. The morphology of the bone marrow was assessed via light microscopy (Leica DMLB, Leica, Germany), and megakaryocytes were counted by an observer blinded to the sample groups. For WT mice, no less than 10 visions were observed in each sample, whereas for TPO^−/−^ and c-mpl^−/−^ mice the megakaryocytes were counted throughout the whole slice of the samples.

### Cell culture

Mouse fetal livers were collected at E14, cut to pieces, digested with 0.125 % Tryspin (Life, Thermo fisher scientific, Waltham, MA, USA), and then filtered successively through 200-mesh (pore size, 74 μm) and 400-mesh (pore size, 37 μm) stainless steel screens. The yielded cells were cultured in RPMI1640 (Gibco, Thermo Fisher scientific, Waltham, MA, USA) supplemented with 10 % FBS (HyClone, Logan, Utah, USA) and incubated at 37 °C in 5 % CO_2_.

### Flow cytometry

Cultured fetal liver cells were stained with PE-CD61 (104308, 1:100, Biolegend, San Diego, CA), FITC-CD41 (13903, 1:200, BioLegend, San Diego, CA), or 7-AAD viability staining solution (420403, 1:20, BioLegend, San Diego, CA) according to instructions provided by the manufacturer. Briefly, about one million cells were collected and resuspended in 100 μL phosphate-buffered saline (PBS) and then stained with respective antibodies according to its recommended usage for 30 min at 4 °C. Then the cells were centrifuged at 1000 rpm for 5 min, and the supernatant was discarded. Then the cells were washed in 1 mL PBS twice before being resuspended in 500 μL PBS. FACS analysis was performed with a flow cytometer (FACSCalibur, BD, USA). More than 10,000 cells were acquired and analyzed by CELLQuest. For each experiment of this part, fetal liver cells isolated from at least three individual mice were used and for at least three repetitions for each group. The operator of the flow cytometer was blinded to the sample groups.

### Administration of H_2_S and TPO

NaHS (Sigma-Aldrich, St. Louis, MO, USA) was applied as a donor of H_2_S [45] in the present study. For the in vivo administration, NaHS and recombinant murine TPO (R&D, NASDAQ, USA) were freshly diluted in sterile saline or PBS for each administration. A single dose of TPO (15 μg kg^−1^) was given by intraperitoneal injection after radiation exposure whereas NaHS was injected once a day during the experimental period. For in vitro experiments, recombinant murine TPO (PeproTech, NJ, USA) was freshly diluted with cell culture medium and added to the cultured cells with a single dose of 10 ng mL^−1^ given at the first day of the experiments. NaHS was diluted with cell culture medium and added to the cultured cells every 8 h.

### Scanning electron microscopy

Specimen were first double fixed with 2.5 % glutaraldehyde and 1 % osmium tetroxide and then gradiently dehydrated with ethanol followed by dehydration in HCP-2 critical point dryer (Haitchi, Tokyo, Japan) with liquid CO_2_. The dehydrated specimen was coated with gold palladium and then observed in a JSM-6360LV scanning electron microscope (JEOL, Tokyo, Japan).

### Confocal microscopy

Cultured fetal liver cells were stained with PE-CD61 (12-0611, 1:40, eBioscience, San Diego, CA), FITC-CD41 (11-0411, 1:200, eBioscience, San Diego, CA), and the DAPI staining solution (C1006, 100 μL, Beyotime, China) and then examined with a confocal laser scanning microscope LSM 710 (Zeiss, Jena, Germany).

### Measurement of plasma H_2_S

Plasma H_2_S levels in some mice were measured according to a method previously described [61]. The concentrations were determined using the standard curve generated with a standard NaHS solution.

### Measurement of plasma TPO

Plasma TPO levels in some mice were measured using the Mouse Thrombopoietin Quantiken ELISA Kit (R&D, NASDAQ, USA) according to the manufacturer’s instructions.

### Statistical analysis

Results were expressed as the mean ± standard error. Unless specifically stated, the data show normal distribution. For two groups, independent-sample *t* test was used. For more than two groups, statistical analysis was performed with one-way ANOVA followed by LSD if the variance is homogeneous or Tamhane’s T2 test when the variance is not homogeneous. For the fecal occult blood assay, the data were transformed in rank cases first and then analyzed with one-way ANOVA as above. Comparisons of mortality were performed with Kaplan-Meier survival curves, in which log-rank test was used to evaluate differences in survival. *P* < 0.05 was considered as statistically significant.

## Additional files

Additional file 1: Figure S1.
**Survival analysis of c-mpl** ^**−/−**^ **and TPO** ^**−/−**^ **mice after exposure to radiation and treated with vehicle.** TPO^**−/−**^ mice after exposure to 5.0 Gy 137Cs then treated with vehicle had a significantly decreased survival compared with that in c-mpl^**−/−**^ mice .***P* < 0.01, log-rank test. (PDF 44.1 kb) Additional file 2: Figure S2.
**H**_**2**_ **S increases circulating leukocytes in WT mice exposed to radiation.** (**a**) NaHS treatment did not change circulating erythrocytes at dosages of 10, 20, 50, 100, and 150 μmol kg^**−**1^ day^**−**1^ in WT mice exposed to radiation at day 7 and day 14. (**b**) NaHS treatment increased circulation leukocytes at a dosage of 100 μmol kg^**−**1^ day^**−**1^ in WT mice exposed to radiation at day 14 but not day 7. Data in the graphs are means ± SEM. *P* values less than 0.05 represent statistical significance. (PDF 311 kb)

## Abbreviations

AA: aplastic anemia
EDTA: ethylenediaminetetraacetic
H_2_S: hydrogen sulfide
MDS: myelodysplastic syndrome
NaHS: sodium hydrosulfide
TBI: total body irradiation
TPO: thrombopoietin
