# Supplementary material for: H2S protects against fatal myelosuppression by promoting the generation of megakaryocytes/platelets
Source: J Hematol Oncol. 2016 Feb 24;9:13. doi: 10.1186/s13045-016-0244-7 (PMC4766725; doi:10.1186/s13045-016-0244-7)
Supplement: Additional file 1: Figure S1. — Survival analysis of c-mpl −/− and TPO −/− mice after exposure to radiation and treated with vehicle. TPO−/− mice after exposure to 5.0 Gy 137Cs then treated with vehicle had a significantly decreased survival compared with that in c-mpl−/− mice .**P < 0.01, log-rank test. (PDF 44.1 kb) [file 13045_2016_244_MOESM1_ESM.pdf]

Additional file 1

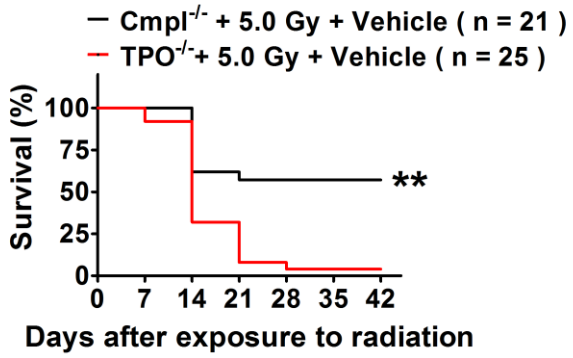

Figure S1. Survival analysis of C-mpl$^{-/-}$ and TPO$^{-/-}$ mice after exposure to radiation and treated with vehicle. TPO$^{-/-}$ mice after exposure to 5.0 Gy ^{137}Cs then treated with vehicle had a significantly decreased survival compared with that in C-mpl$^{-/-}$ mice .***P* < 0.01, log-rank test.
