# Supplementary material for: H2S protects against fatal myelosuppression by promoting the generation of megakaryocytes/platelets
Source: J Hematol Oncol. 2016 Feb 24;9:13. doi: 10.1186/s13045-016-0244-7 (PMC4766725; doi:10.1186/s13045-016-0244-7)
Supplement: Additional file 2: Figure S2. — H 2 S increases circulating leukocytes in WT mice exposed to radiation. (a) NaHS treatment did not change circulating erythrocytes at dosages of 10, 20, 50, 100, and 150 μmol kg−1 day−1 in WT mice exposed to radiation at day 7 and day 14. (b) NaHS treatment increased circulation leukocytes at a dosage of 100 μmol kg−1 day−1 in WT mice exposed to radiation at day 14 but not day 7. Data in the graphs are means ± SEM. P values less than 0.05 represent statistical significance. (PDF 311 kb) [file 13045_2016_244_MOESM2_ESM.pdf]

Additional file 2

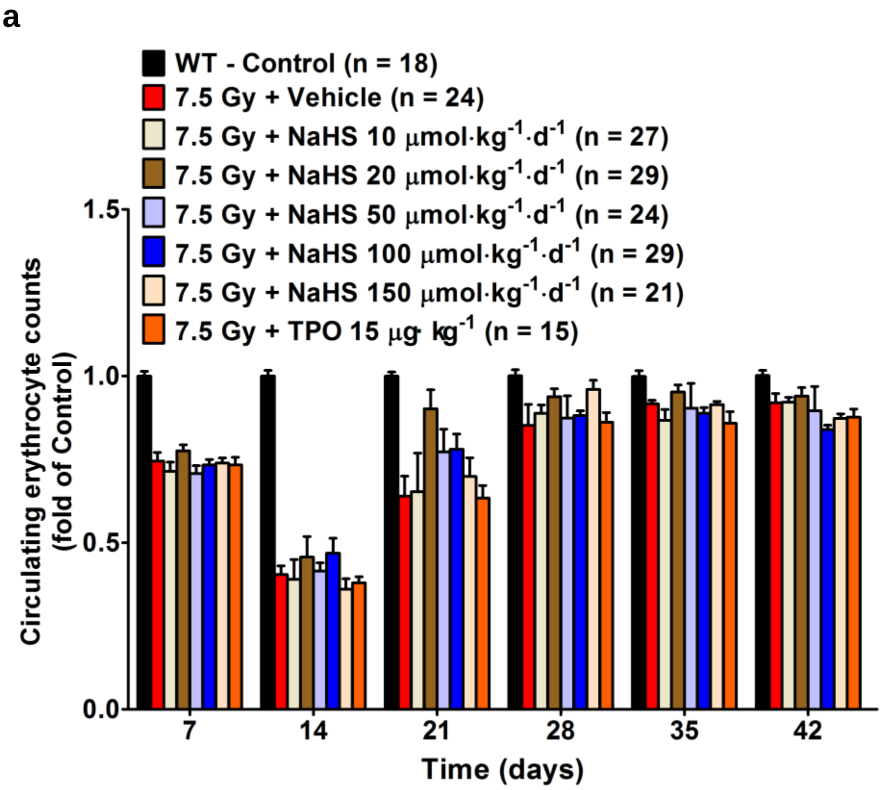

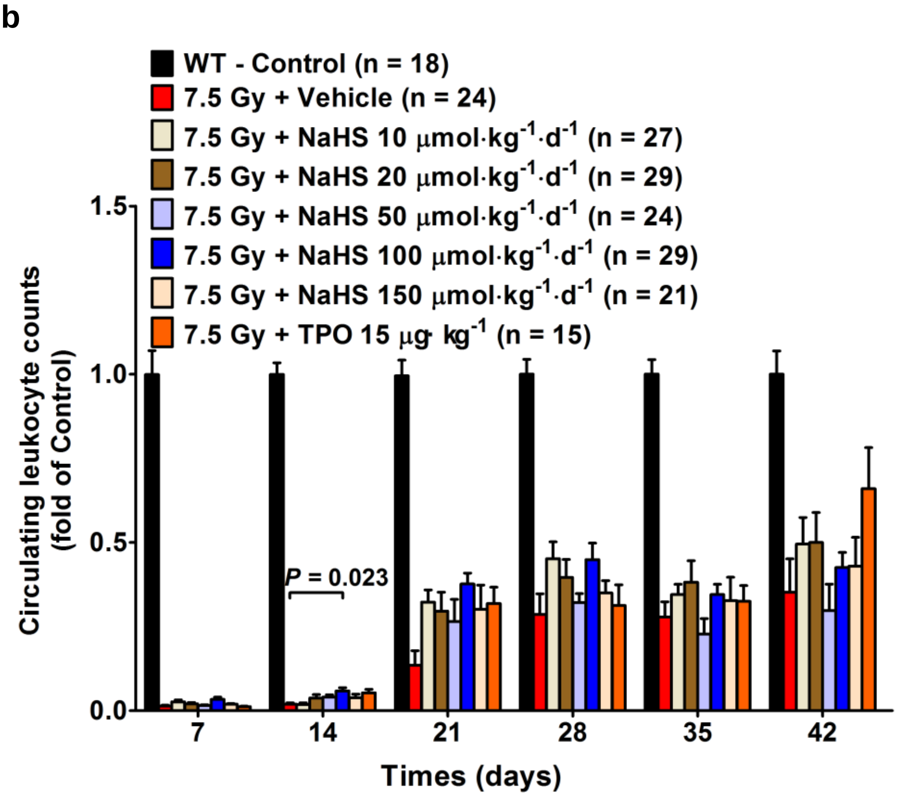

Figure S2. H_2S increases circulating leukocytes in WT mice exposed to radiation. (**a**) NaHS treatment did not change circulating erythrocytes at dosages of 10, 20, 50, 100 and 150 $\mu mol \cdot kg^{-1}$ d^{-1} in WT mice exposed to radiation at day 7 and day 14. (**b**) NaHS treatment increased circulation leukocytes at a dosage of 100 $\mu mol \cdot kg^{-1}$ d^{-1} in WT mice exposed to radiation at day 14 but not day 7. Data in the graphs are means ± SEM. *P* values less than 0.05 represent statistical significance.
